# Diversity of Genetic and Vegetative Compatibility Group of *Colletotrichum coccodes* Isolates from Chile Using Amplified Fragment Length Polymorphism Markers

**DOI:** 10.3390/jof10030200

**Published:** 2024-03-06

**Authors:** Kholoud M. Alananbeh, Viviana Rivera, Ivette Acuña Bravo, Gary Secor, Neil C. Gudmestad

**Affiliations:** 1 Department of Plant Protection, School of Agriculture, The University of Jordan, Amman 11942, Jordan; 2 Department of Plant Pathology, North Dakota State University, Fargo, ND 58108, USA; viviana.rivera@ndsu.edu (V.R.); gary.secor@ndsu.edu (G.S.); neil.gudmestad@ndsu.edu (N.C.G.); 3 INIA Remehue-Ruta 5, Osorno 5290000, Chile; iacuna@inia.cl

**Keywords:** vegetative compatibility groups, cryptic sexual cycle, geographic origin, population biology, linkage disequilibrium

## Abstract

*Colletotrichum coccodes* (Wallr.) Hughes is an asexual fungus with five vegetative compatibility groups. It was postulated that *C. coccodes* was isolated at the center of origin of potato at one time, and due to the movement of potato around the globe, the fungus was established on each continent but became bottlenecked and genetically unable to form stable heterokaryons via vegetative compatibility grouping (VCG) studies. The objectives of this study were (i) to determine if the VCGs around the world are related to the VCGs in Chile, (ii) to determine the diversity of *C. coccodes* populations in Chile, and (iii) to find any evidence for a cryptic sexual life cycle for this fungus. Worldwide *C. coccodes* populations have been found to be genetically correlated and belong to one or more *C. coccodes*-identified VCGs. The most distributed VCG in Chile was VCG2, which is the most common VCG in North America. We hypothesize that one or more VCGs had spread from Chile to the rest of the world. Precautions and further studies should be investigated by using other molecular markers and gene sequencing.

## 1. Introduction

*Colletotrichum coccodes* (Wallr.) Hughes is a cosmopolitan imperfect fungus that causes black dot in potato, a serious blemish disease with a worldwide distribution [1,2]. It is also involved in the early dying complex of potato, capable of causing root and belowground degradation [3,4]. On culture media, *C. coccodes* is characterized by its white aerial sparse mycelium, abundant black sclerotia, and acervuli production [5,6]. *C. coccodes* exchanges its genetic materials through anastomosis with another fungal strain from the same group, a process called vegetative compatibility. Through this process, asexual fungi can be placed into similar clusters called vegetative compatibility groups (VCGs) [7]. Isolates that form stable heterokaryons after anastomosis are considered to be identical and are regarded as belonging to the same VCG [7]. Many VCGs have been identified for *C. coccodes* consisting of multiple populations from different geographic origins, i.e., North America, Europe, and Australia [8,9,10,11]. Both traditional [8,9,11] and molecular [12,13,14] methods have been used to efficiently differentiate these VCGs. However, many limitations have been reported for traditional techniques [15,16]. Analysis of VCGs using amplified fragment length polymorphism (AFLP) analysis was found capable of differentiating the North American *C. coccodes* population into five groups, almost completely agreeing with the traditional VCG method [12]. Using AFLP analysis, NA-VCG4 and NA-VCG5, and NA-VCG6 and NA-VCG7 could not be separated, because of their high genetic similarity [13]. A global study conducted to determine the relationship of *C. coccodes* populations originating from different geographic origins demonstrated that the global population is closely related at the molecular genetics level. However, geographic isolation was significant [13], a reason that could be speculated for vegetative incompatibility [10] or limited complementation among isolates of *C. coccodes* originating from different continents [8].

For the last 60 years, the center of origin of potato was hypothesized to be from the Andes. However, a recent molecular study [17] presented the first evidence that lowland Chile is the center of origin of potato. It is postulated that the *C. coccodes* population was isolated at the center of origin of potato at one time, and due to the movement of potato around the globe, the fungus became established on each continent. However, due to the lack of intermixing, *C. coccodes* populations on a continent became bottlenecked. This would likely occur when a small population of *C. coccodes* is introduced into a continent; any genetic drift can occur quickly, thereby reducing genetic variation due to the small population size. The main objectives of this study were (i) to determine if selected *C. coccodes* isolates from across the globe are related to isolates of this fungus that originate from the center of origin of potato using amplified fragment length polymorphism markers (AFLP), (ii) to determine the diversity of *C. coccodes* populations in Chile, and (iii) to determine if there is evidence for a cryptic sexual life cycle for this asexual fungus.

## 2. Materials and Methods

### 2.1. Colletotrichum coccodes Isolates

A total of 148 *C. coccodes* isolates were used in this study (Table 1). Of the 148 isolates, there were 99 isolates from Chile representing four locations (Chiloe Island, Osorno, La Union, and Puerto Montt) (Table S1). These isolates were recovered from 14 potato cultivars (Clavela, Desirée, Michuña negra, Michuña roja, Murta, Pie, Pukará, Romano, Asterix, Cardinal, Karú, Patagonia, Yagana, and Rosara). Twenty isolates previously characterized from the North American *C. coccodes* population were also used [12]. Additionally, 13 tester isolates representing the seven NA-VCGs were included. Five, three, and eight isolates from Australia [8], South Africa (Lahkim, T (Unpublished)), and Europe [9,11], respectively, were included. These isolates were previously assigned into their VCGs based on AFLP analysis [13]. For the Chilean population, isolates were collected and preserved as frozen colonized silica gel crystals [18]. Isolates were plated on potato dextrose agar and visually examined to ensure they were free from contamination [19].

### 2.2. DNA Extraction

*Colletotrichum coccodes* isolates were grown in Richard’s solution [20] and processed as previously described [13]. Cetyltrimethyl ammonium bromide (CTAB) [21] was used for DNA extraction. DNA quantity and purity were assessed using a Nanodrop 2000c (Thermo Fisher Scientific Inc., Waltham, MA, USA) and agarose gel visualization. DNA quantities were diluted to a 100 ng/μL concentration.

### 2.3. Molecular Confirmation

*Colletotrichum coccodes* isolates from Chile were confirmed for species using Cc1NF1 and Cc2nR1 primers [22]. *C. coccodes* tester strain (C501, NA-VCG7) and blank water were used as positive and negative controls, respectively. Internal transcribed spacer (ITS) region [23] was amplified for 143 isolates (Australia, 5; Chile, 96; Europe, 8; South Africa, 1; USA; and its testers, 33) (Table S1). All Chilean isolates used in this study yielded the 349 bp polymorphic DNA band [22]. Sequences were edited by BioEdit Sequence Alignment Editor software 95/98/NT [24] to create the consensus sequence from the forward and reverse sequences and aligned using MultiAlin [25] and BLASTn [26] on http://www.ncbi.nlm.nih.gov (accessed on 5 August 2013). Sequences were submitted to the Gene Bank and accession numbers JX293923–JX294064 were obtained (Table S1).

### 2.4. AFLP Assays

AFLP assays [27] were performed using AFLP kit (Li-Cor, Lincoln, NE, USA) according to the manufacturer’s instructions. Template DNA was digested with *Eco*RI and *Mse*I restriction enzymes, ligated with adapters, and pre-amplified using *Eco*RI and *Mse*I primers. Three selective primers [12] labeled with fluorescent dyes 6-FAM, HEX, and NED were used for selective amplification. Products were separated using automated DNA sequencer (3130xl Genetic Analyzer; Applied Biosystems, Waltham, MA, USA). Data analysis was performed using Genemapper Software Version 4.0 (Applied Biosystems, USA). To ensure reproducibility of the AFLP results, 16 DNA samples were digested, ligated, pre-amplified, and amplified three times each. Blank water was also used as the negative control.

### 2.5. Cluster Analysis

Binomial data was created by combining the three selective amplifications reactions. Data was analyzed and cluster analysis was performed using WinBoot [28] with 1000 bootstraps. Principal coordinate analysis (PCoA) was also performed for the 135 *C. coccodes* isolates using GenAlex 6.3 [29].

### 2.6. Population Genetics Statistics

Data generated from the 456 loci were analyzed using GenAlex 6.3 software [29], POPGENE version 1.32 [30], and Multilocus 1.3 b software [31]. The number and percentage of polymorphic loci, Nei’s gene diversity (h), number of distinct genotypes (G), genotypic diversity (GD), linkage disequilibrium (LD), number of different bands, number of bands unique to a single population, population differentiation across the loci, analysis of molecular variance (AMOVA), and genotypic diversity versus number of loci were all calculated. AMOVA was conducted for the 135 *C. coccodes* as a global population; for isolates from Chile, analysis was based on both the locations and potato cultivars from which the isolates were obtained, and on the clusters obtained through cluster analysis. The variance was partitioned into two covariance components of Phi (Ф) fixation indices (ФRT and ФPT) with a level of significance of *p* < 0.05 and 1000 permutations [32].

## 3. Results

### 3.1. AFLP Analysis

The three selective amplification primers used in this study generated 456 putative loci, 143 loci for *Eco*RI-AC/*Mse*I-C, 152 for *Eco*RI-AG/*Mse*I-C, and 161 for *Eco*RI-AT/*Mse*I-C. E*co*RI-AC/*Mse*I-C had 97.2% of polymorphism and a gene diversity of 0.30, *Eco*RI-AG/*Mse*I-C had 98.68% of polymorphism and a gene diversity of 0.31, and *Eco*RI-AT/*Mse*I-C had 98.76% of polymorphism and a gene diversity of 0.30. The three primer set data were combined for further analysis due to their close values of gene diversity and percentage of polymorphic loci.

### 3.2. Cluster Analysis

**Figure 1:**
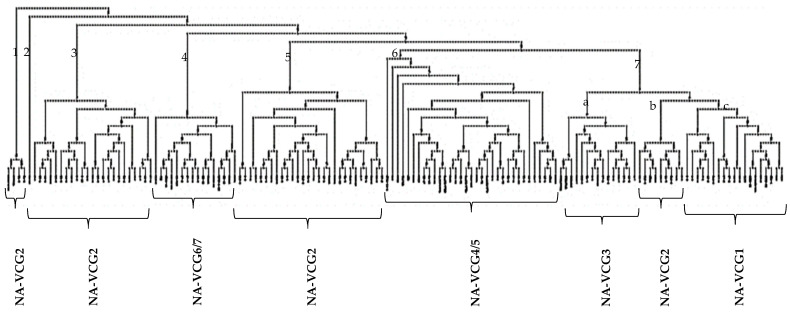
Cluster analysis of the 135 isolates of *C. coccodes* from five different regions and 13 NA-VCG tester strains. Dendogram was generated through WINBOOT software 6.1 with 1000 bootstraps. Cluster analysis was generated based on an isolate’s relationship to a known NA-VCGs ^a^. ^a^ Detailed information pertaining to isolate names, testers, and NA VCG assignments is provided in Supplementary Materials (Figure S1).

Cluster analysis based on 1000 bootstraps and UPGMA separated *C. coccodes* isolates into seven clusters (Figure 1, Figure S1). The differentiation among the seven clusters was very high (0.43) (Table 2). Isolates were assigned based on their clustering with known NA-VCG tester strains. Cluster 7c had isolates belonging to NA-VCG1. This cluster had 18 isolates: 13 from Chile, 3 from the USA, and 2 from Europe (Table 3). Clusters 1, 2, 3, 5, and 7b belonged to NA-VCG2 (Figure 1). In this cluster, there were 63 isolates: 58 from Chile and 5 from the USA (Table 3). Clusters 2 and 3 had isolates only from Chile but showed the highest similarity (88%) to NA-VCG2. Fourteen isolates belonging to NA-VCG3 were found in cluster 7a, including twelve from Chile and two from the USA. For NA-VCG4/5, 24 isolates were assigned to cluster 6. It included nine isolates from Chile, three from the USA, five from Australia, two from South Africa, and five from Europe (Table 3). Finally, for NA-VCG6/7, the very distinctive group, 16 isolates were assigned to cluster 4. This group included seven isolates from Chile, seven from the USA, one from South Africa, and one from Europe (Table 3).

Excluding the tester isolates, NA-VCG2 had the highest number of isolates for the Chilean population, containing 58 out of the 99 isolates, followed by NA-VCG1 and NA-VCG3. The NA-VCG6/7 group had the lowest number of isolates from Chile (Table 3).

**Figure 2:**
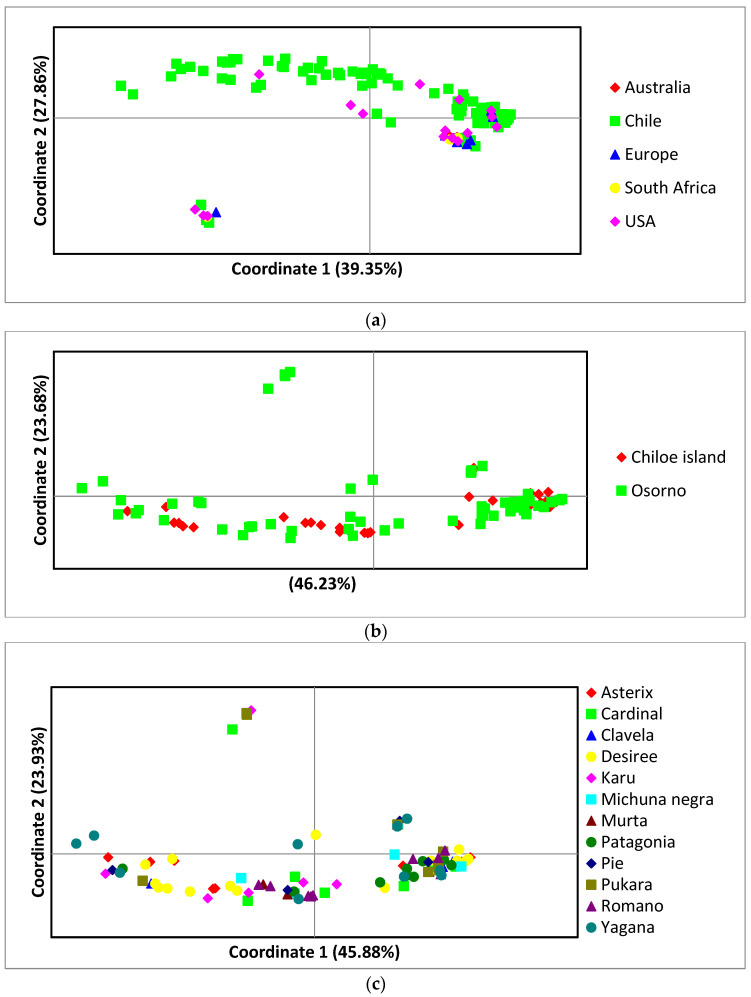
Principal coordinates analysis for 135 *C. coccodes* isolates. (**a**) Global population (*n* = 135), (**b**) two locations in Chile, and (**c**) 12 potato cultivars in Chile. For the global population, coordinate axis 1 explains 39.35% of the variation, coordinate axis 2 explains 27.86% of the variation, and coordinate 3 explains 18.98% of the variation. For the two Chilean locations, coordinate axis 1 explains 46.23% of the variation, coordinate axis 2 explains 23.68% of the variation, and coordinate 3 explains 12.34% of the variation. For the 12 Chilean potato cultivars, coordinate axis 1 explains 45.88% of the variation, coordinate axis 2 explains 23.93% of the variation, and coordinate 3 explains 12.38% of the variation.

For principal coordinate analysis, the first three axes explained most of the variability in the *C. coccodes* population studied for the global population, the 2 Chilean locations, and 12 potato Chilean cultivars (Figure 2). The isolates belonging to NA-VCG6/7 were distinct from the rest of the VCGs in the three PCoA analyzed (Figure 2) and are consistent with UPGMA cluster analysis (Figure 1).

### 3.3. Population Genetics Statistics of C. coccodes Populations

Considering *C. coccodes* as one population (*n* = 135), both total genetic diversity (Ht = 0.26) and gene diversity within a regional population (Hs = 0.22) were relatively high. These values were similar to the values obtained when merging the three AFLP selective primer pair data. The number of polymorphic loci was high (*n* = 449) for the 135 *C. coccodes* isolates (Table 4). The gene diversity (h) ranged from 0.02 in Australia to 0.31 in the USA. *C. coccodes* isolates from the two locations and the 12 potato cultivars in Chile also showed high gene diversity. Gene diversity was 0.25 and 0.29 for Chiloe Island and Osorno locations and ranged from 0.0.18 to 0.29 for Murta and Pukara cultivars, respectively (Table 4). There was a high number of distinct genotypes in all the *C. coccodes* populations tested (*G* = 122). Genotypic diversity was also high, and it ranged between 0.95 and 1.00% in all the populations (Table 4). Linkage disequilibrium values were low for the *C. coccodes* populations but was significantly different from zero (*p* < 0.01), suggesting no random association of alleles in either of these populations. However, Australian, European, and South African isolates showed very high LD values. This can be attributed to the low number of isolates used from these regions.

As a global population, Chile and USA populations had 15 and 21 unique bands, respectively. Based on two locations in Chile, Osorno had a high number of unique bands (59) compared to Chiloe Island (7) (Table 4). Similarly, the *C. coccodes* population obtained from the potato cultivar, Asterix, had five unique bands compared to the other 13 cultivars. The differentiation among the five global populations (Gst = 0.22) and the two locations in Chile (Gst = 0.26) was relatively high. However, the differentiation among the 12 cultivars was highest (Gst = 0.426).

AMOVA showed that there was 13% of variation that originated from variations between geographic origins (ФPT, *p* = 0.006), 1% of variation between the two Chilean locations (*p* = 0.203), and 2% between the 12 Chilean potato cultivars studied (*p* = 0.043) (Table 2).

Pairwise comparisons of population genetic identity and genetic distance among the five geographic regions (Australia, Chile, Europe, South Africa, and the USA) and for the four Chilean populations (Chiloe Island, La Union, Osorno, and Peurto Montt) were not reliable, due to the unbalanced sample size among them, and are thus not presented or discussed. For potato cultivars, genetic similarity values ranged from 0.74 between Karu and Michuna Roja cultivars to 0.98 between Asterix and Desiree cultivars (Table 5).

## 4. Discussion

This study was conducted to determine the similarity of the global population of *C. coccodes* isolates to those obtained from the Chilean population using AFLP markers. Ninety-nine isolates representing four locations and 12 cultivars in Chile were used. AFLP markers could differentiate and cluster the isolates into their presumptive VCGs. However, not every VCG was found in distinct clusters, i.e., VCG2 was found in four clusters. The three selective AFLP primers were polymorphic and informative, confirming the results from previous studies [12,13,14].

Previous studies suggested that *C. coccodes* populations are distinct by continent [9,10,11] based on nitrate nonutilizing mutant vegetative compatibility grouping. However, a very small number of isolates from North America, Australia, and Europe successfully anastomosed [8]. *C. coccodes* populations from four regions (Australia, North America, Europe, and South Africa) showed that these populations intermingled with the North American population, but geographic isolation was likely the reason that they could be distinguished since they formed distinct sub-clusters within the main clusters [13]. That study speculated that the global *C. coccodes* is genetically similar, and to demonstrate that, isolates from Chile were used here. The isolates from Chile clustered successfully with the North American and the global population. Interestingly, the Chilean population was 96% genetically similar to the North American population, a population that had most of its isolates belonging to NA-VCG2 [13,14]. Similarly, the Chilean population had its highest number of isolates (*n* = 58) belonging to NA-VCG2.

Interestingly, the Chilean population has a high genetic diversity among locations and among potato cultivars. The Andean origin hypothesis for European potato has been widely accepted over the last 60 years; however, all the modern potato cultivars have Chilean germplasm attributed to breeding activities involving Chilean landraces. Asexual fungi are expected to have low genotypic diversity [33]. In our study, gene diversity, genotypic diversity, and the number of distinct genotypes were very high and similar to the values found in the global populations of *C. coccodes* from Australia, Europe, North America, and South Africa [13]. This suggests either a possibility of a cryptic sexual cycle [34] for *C. coccodes* and/or a high level of gene flow due to the introduction of the pathogen [35]. The significant linkage disequilibrium values obtained here and in the previous studies [13] support the hypothesis of a cryptic sexual cycle in *C. coccodes*. However, many factors should be considered when suggesting sexual reproduction such as admixtures of differentiated genetic isolates, bottlenecks, rapid population expansion, and AFLP homoplasy [34]. However, a cryptic life cycle is not new for asexual fungi. Previous population genetic studies provided the existence of sexual reproduction [36], recombination [37,38,39], and parasexuality in asexual fungi [40]. The hypothesized cryptic sexual cycle should be further investigated by other molecular markers and gene sequencing. Additionally, geographic isolation that can cause genetic differentiation among the global regions would be expected to create less genotype flow [13], which also supports the possibility for a cryptic sexual life cycle.

As previously postulated, the *C. coccodes* population was isolated at the potato center of origin at one time and the fungus became established on each continent when the potato was disseminated. Limited genetic diversity and rapid spread are typical conditions for considering any pathogen as clonal and introduced [35]. For example, *Phytophthora cinnamomi* in Australia and South Africa and *P. ramorum* in the USA and Europe were considered introduced pathogens with low genetic diversity [41,42,43]. Chilean *C. coccodes* populations are characterized by their high genetic diversity and the existence of the five VCGs that were previously characterized by AFLP analysis [12,13] with a high number of isolates belonging to NA-VCG2. Similarly, the USA has a high gene diversity and VCG diversity compared to the Chilean *C. coccodes* population. Australian, European, and South African *C. coccodes* isolates belong to Na-VCG4/5 and NA-VCG6/7, and they had low gene diversity except for South African isolates due to the low number of isolates studied [13]. These findings support that the *C. coccodes* population is genetically similar (96%) to the USA population. These findings support the possibility for a cryptic life cycle in Chile and the USA and/or high gene flow among South and North America that caused the high gene diversity.

As previously reported, AFLPs proved to be an effective means to study the global diversity of *C. coccodes*. However, using more selective primers, co-dominant markers such as SSRs and/or multiple gene and multilocus phylogenetics sequencing would provide greater insights into the phylogeny and population biology of this fungus. Future work will concentrate on genotyping by sequencing and phylogenetic analysis to determine whether multiple gene sequencing could efficiently differentiate the different *C. coccodes* VCGs. Additionally, this study confirms the potential of identifying durable resistance to *C. coccodes* that would be globally useful in managing black dot of potato. Additionally, NA-VCG2 and NA-VCG4/5 have high genetic similarity, so selecting durable resistance against one of these groups could be effective on a wide scale [13].

In conclusion, the global population of *C. coccodes* is genetically correlated and belongs to one or more of these fungus identified VCGs: VCG1, VCG2, VCG3, VCG4/5, and VCG6/7. The results of this study strongly suggest that the Chilean *C. coccodes* population has high genetic diversity within its population. The most distributed VCG in Chile was VCG2, which is similar to the most common VCG in North America. In comparison, previous studies determined that VCG4/5 was the most common VCG in Europe and Australia [13].

## Figures and Tables

**Table 1:** *Colletotrichum coccodes* isolates from different geographic origins used in this study.

Country	Location	Cultivar	No. Isolates
Chile, South America ^1^	Coñab, Achao, Chiloe Island	Clavela	3
	Coñab, Achao, Chiloe Island	Desirée	9
	Coñab, Achao, Chiloe Island	Michuña negra	5
	Coñab, Achao, Chiloe Island	Michuña roja	1
	Coñab, Achao, Chiloe Island	Murta	3
	Coñab, Achao, Chiloe Island	Pie	5
	Coñab, Achao, Chiloe Island	Pukará	9
	Coñab, Achao, Chiloe Island	Romano	9
	Remehue, Osorno	Asterix	10
	Remehue, Osorno	Cardinal	8
	Remehue, Osorno	Desirée	6
	Remehue, Osorno	Karú	7
	Remehue, Osorno	Patagonia	9
	Remehue, Osorno	Yagana	13
	Rapaco, La Union	Desirée	1
	Nueva Braunau, Puerto Montt	Rosara	1
USA, North America ^2^			20
Australia ^3^			5
South Africa			3
Europe			8
Testers ^4^			13
Total			148

^1^ Isolates were collected in the Lake District of Chile in 2010. ^2^ The isolates were chosen based on Heilmann et al. (2006) [12]. ^3^ The isolates were chosen based on Alananbeh et al., 2014 [13]. ^4^ The isolates represent the seven North American VCG testers.

**Table 2:** Analysis of molecular variance (AMOVA) for *C. coccodes* populations.

Source of Variation	df	Est. Var. ^a^	%	Ф Value	*p* (Rand ≥ Data) ^b^
Based on geographic origin ^c^					
Among populations (ФPT) ^d^	4	9.99	13%	0.134	0.006
Within populations	130	64.80	87%		
Chile population					
a. Based on location					
Among locations (ФPT)	1	0.34	1	0.006	0.203
Within locations	95	65.94	99		
b. Based on cultivar					
Among cultivars (ФPT)	11	1.46	2	0.022	0.043
Within cultivars	85	64.67	98		
For clusters					
Among clusters (ФPT)	4	34.38	43	0.434	0.001
Within clusters	130	44.8	57		

^a^ Estimated variation using GenAlex 6.3 software. ^b^ Probability of obtaining low Phi values was determined by 1000 permutations. ^c^ *C. coccodes* was partitioned into five populations (the USA, Australia, Europe, and South Africa) and four regions (America, South Africa, Australia, and Europe). The sample size of these geographic populations was unbalanced. ^d^ PhiPT was calculated as the proportion of estimated variance among populations, relative to the total estimated variance.

**Table 3:** Assigning of the 135 *C. coccodes* isolates into their VCGs based on clustering with known NA-VCG tester strains.

VCG	Geographic Origin					
	Chile	USA	Australia	South Africa	Europe	Total
NA-VCG1	13	3	0	0	2	18
NA-VCG2	58	5	0	0	0	63
NA-VCG3	12	2	0	0	0	14
NA-VCG4/5	9	3	5	2	5	24
NA-VCG6/7	7	7	0	1	1	16
Total	99	20	5	3	8	135

**Table 4:** Genetic variation statistics for the 456 loci from *C. coccodes* isolates based on geographic differentiation among continents, two locations, and 12 potato cultivars in Chile.

Country		Sample Size	No. Polymorphic Loci ^a^	% ^a^	h ^b^	G ^c^	GD ^d^	LD ^e^	No. Unique Bands ^f^
One population ^h^		135 ^g^	449	98.46	0.21	122	0.99	0.06	-
Australia		5	26	5.70	0.02	4	0.90	0.27	0
Europe		8	283	62.06	0.19	8	1	0.29	0
South Africa		3	206	45.18	0.20	3	1	0.76	0
USA, North America		20	420	92.11	0.31	16	0.97	0.08	21
Chile, South America		99	426	93.42	0.28	94	0.99	0.06	15
Based on location	Chiloe island	35	365	80.04	0.25	35	1	0.06	7
	Osorno	62	418	91.67	0.29	57	0.99	0.06	59
	La Union	1	-	-	-	-	-	-	-
	Puerto Montt	1	-	-	-	-	-	-	-
Based on cultivar	Asterix	10	345	75.66	0.28	10	1	0.08	5
	Cardinal	8	313	68.64	0.24	7	0.96	0.13	0
	Clavela	3	209	45.83	0.20	3	1	0.30	1
	Desiree	16	341	74.78	0.26	16	1	0.09	1
	Karu	7	308	67.54	0.27	6	0.95	0.17	0
	Michuna negra	5	242	53.07	0.22	5	1	0.03	1
	Michuna roja	1	-	-	-	-	-	-	-
	Murta	3	189	41.15	0.18	3	1	-0.002	0
	Patagonia	9	275	60.31	0.21	9	1	0.09	1
	Pie	5	291	63.82	0.25	5	1	0.06	0
	Pukara	9	373	81.80	0.29	8	0.97	0.12	0
	Romano	9	271	59.43	0.20	9	1	0.09	0
	Rosara	1	-	-	-	-	-	-	-
	Yagana	13	325	71.27	0.27	11	0.97	0.12	1

^a^ The percentage of polymorphic loci; ^b^ h: Nei’s gene diversity; ^c^ *G*: number of distinct genotypes; ^d^ *GD*: genotypic diversity; ^e^ measurement of linkage disequilibrium (LD), all values were significant from zero (*p* < 0.01) except for Murta cultivar; ^f^ no. unique bands: no. of bands unique to a single population; ^g^ tester strains were excluded from this analysis; ^h^ all isolates from the different countries were analyzed as one population (*n* = 135) without the tester isolates.

**Table 5:** Pairwise comparison matrix of Nei genetic identity (above the asterisks) and Nei genetic distance (below the asterisks) for the 135 *C. coccodes* populations from 14 potato cultivars in Chile.

Potato Cultivar	Asterix	Cardinal	Clavela	Desiree	Karu	Michuna Negra	Michuna Roja	Murta	Patagonia	Pie	Pukara	Romano	Rosara	Yagana
Asterix	****	0.95	0.93	0.98	0.93	0.95	0.82	0.95	0.95	0.97	0.94	0.96	0.83	0.97
Cardinal	0.06	****	0.92	0.95	0.94	0.93	0.81	0.93	0.95	0.94	0.94	0.95	0.84	0.95
Clavela	0.07	0.08	****	0.94	0.88	0.92	0.83	0.92	0.93	0.93	0.91	0.93	0.84	0.92
Desiree	0.02	0.05	0.06	****	0.93	0.95	0.81	0.95	0.95	0.96	0.93	0.96	0.83	0.97
Karu	0.07	0.06	0.13	0.07	****	0.89	0.74	0.90	0.89	0.91	0.92	0.90	0.76	0.93
Michuna Negra	0.05	0.07	0.09	0.06	0.12	****	0.81	0.94	0.96	0.96	0.94	0.93	0.88	0.96
Michuna Roja	0.20	0.21	0.19	0.21	0.30	0.21	****	0.78	0.86	0.86	0.82	0.85	0.77	0.78
Murta	0.05	0.07	0.09	0.05	0.11	0.07	0.25	****	0.95	0.93	0.93	0.95	0.86	0.94
Patagonia	0.06	0.05	0.07	0.06	0.11	0.05	0.16	0.05	****	0.96	0.95	0.95	0.91	0.94
Pie	0.03	0.06	0.07	0.04	0.09	0.04	0.15	0.07	0.04	****	0.94	0.95	0.84	0.96
Pukara	0.06	0.06	0.10	0.07	0.08	0.06	0.20	0.07	0.05	0.06	****	0.93	0.88	0.94
Romano	0.04	0.05	0.07	0.04	0.10	0.07	0.17	0.05	0.05	0.05	0.07	****	0.85	0.93
Rosara	0.19	0.17	0.18	0.18	0.27	0.13	0.26	0.15	0.09	0.17	0.13	0.16	****	0.84
Yagana	0.03	0.05	0.08	0.03	0.07	0.04	0.25	0.06	0.06	0.04	0.07	0.07	0.18	****

## Data Availability

Data are contained within the article and Supplementary Materials.

## Supplementary Materials

The following supporting information can be downloaded at https://www.mdpi.com/article/10.3390/jof10030200/s1. Figure S1: Consensus tree for 148 *C. coccodes* isolates used in this study. The tree was executed using WinBoot software with 1000 bootstraps. Table S1. Detailed information for Colletotrichum coccodes isolates amplified using the internal transcribed spacer and their accession numbers on National Center for Biological Information (NCBI).

## References

[1] Lees A.K., Hilton A.J. (2003). Black dot (*Colletotrichum coccodes*): An increasingly important disease of potato. Plant Pathol..

[2] Cano J., Guarro J., Gene J. (2004). Molecular and morphological identification of *Colletotrichum* species of clinical interest. J. Microb..

[3] Johnson D.A., Geary B., Tsror L. (2018). Potato Black Dot—The Elusive Pathogen, Disease Development and Management. Am. J. Potato Res..

[4] Pasche J.S., Taylor R.J., Gudmestad N.C. (2010). Colonization of potato by *Colletotrichum coccodes*: Effect of soil infestation, seed and foliar inoculation. Plant Dis..

[5] McIntyre G.A., Rusanowski C. (1975). Scanning electron microscope observations of the development of sporophores of *Colletotrichum atrementarium* on infected potato periderm. Am. Potato J..

[6] Davis J.R., Johnson D.A., Stevenson W.R., Loria R., Franc G.D., Weingartner D.P. (2002). Diseases caused by fungi—Black dot. Compendium of Potato Diseases.

[7] Leslie J.F. (1993). Fungal vegetative compatibility. Annu. Rev. Phytopathol..

[8] Ben-Daniel B., Bar-Zvi D., Johnson D., Harding R., Hazanovsky M., Tsror Lahkim L. (2010). Vegetative compatibility groups in *Colletotrichum coccodes* subpopulations from Australia and genetic links with subpopulations from Europe/Israel and North America. Phytopathology.

[9] Nitzan N., Hazanovsky M., Tal M., Tsror Lahkim L. (2002). Vegetative compatibility groups in *Colletotrichum coccodes*, the causal agent of black dot on potato. Phytopathology.

[10] Nitzan N., Tsror (Lahkim) L., Johnson D.A. (2006). Vegetative compatibility groups and aggressiveness of North American isolates of *Colletotrichum coccodes*, the causal agent of potato black dot. Plant Dis..

[11] Shcolnick S., Dinoor A., Tsror Lahkim L. (2007). Additional vegetative compatibility groups in *Colletotrichum coccodes* subpopulations from Europe and Israel. Plant Dis..

[12] Heilmann L., Nitzan N., Johnson D.A., Pasche J.S., Doetkott C., Gudmestad N.C. (2006). Genetic variability in the potato pathogen *Colletotrichum coccodes* as determined by Amplified Fragment Length Polymorphism and vegetative compatibility group analyses. Phytopathology.

[13] Alananbeh K.M., Lahkim L.T., Gudmestad N.C. (2014). Genetic Diversity of a Global Population of *Colletotrichum Coccodes* using amplified fragment length polymorphism markers. Am. J. Potato Res..

[14] Alananbeh K.M., Gudmestad N.C. (2016). Genetic diversity of *Colletotrichum coccodes* in the United States using amplified fragment length polymorphism analysis. J. Gen. Plant Pathol..

[15] Strausbaugh C.A., Schroth M.N., Weinhold A.R., Hancock J.G. (1992). Assessment of vegetative compatibility of *Verticillium dahliae* tester strains and isolates from California potatoes. Phytopathology.

[16] Joaquim T.R., Rowe R.C. (1990). Reassessment of vegetative compatibility relationships among strains of *Verticillium dahliae* using nitrate nonutilizing mutants. Phytopathology.

[17] Ames M., Spooner D.M. (2008). DNA from herbarium specimens settles a controversy about origins of the European potato. Am. J. Bot..

[18] Smith D., Kirsop B.E., Snell J.J.S. (2005). Maintenance of Fungi. Maintenance of Microorganisms.

[19] Rivera-Varas V.V., Freeman T.A., Gudmestad N.C., Secor G.A. (2007). Mycoparasitism of *Helminthosporium solani* by *Acremonium strictum*. Phytopathology.

[20] Xu M., Huaracha E., Korban S.S. (2001). Development of sequence characterized amplified regions (SCARs) from amplified fragment length polymorphism (AFLP) markers tightly linked to the Vf gene in apple. Genome.

[21] Doyle J.J., Doyle J.L. (1987). A rapid DNA isolation procedure for small quantities of fresh leaf tissue. Phytochem. Bull..

[22] Cullen D.W., Lees A.K., Toth I.K., Duncan J.M. (2002). Detection of *Colletotrichum coccodes* from soil and potato tubers by conventional and quantitative real-time PCR. Plant Pathol..

[23] White T.J., Bruns T., Lee S., Taylor J.W., Innis M.A., Gelfand D.H., Sninsky J.J., White T.J. (1990). Amplification and direct sequencing of fungal ribosomal RNA genes for phylogenetics. PCR Protocols: A Guide to Methods and Applications.

[24] Hall T.A. (1999). BioEdit: A user-friendly biological sequence alignment editor and analysis program for Windows 95/98/NT. Nucleic Acids Symp. Ser..

[25] Corpet F. (1988). Multiple sequence alignment with hierarchical clustering. Nucleic Acids Res..

[26] Lobo I. (2008). Basic local alignment search tool (BLAST). Nat. Educ..

[27] Vos P., Hogers R., Bleeker M., Reijans M., van de Lee T., Hornes M., Frijters A., Pot J., Peleman J., Kuiper M. (1995). AFLP: A new technique for DNA fingerprinting. Nucleic Acid. Res..

[28] Yab I., Nelson R.J. (1996). WinBoot: A Program for Performing Bootstrap Analysis of Binary Data to Determine the Confidence Limits of UPGMA-Based Dendograms.

[29] Peakall R., Smouse P.E. (2006). GenAlex 6: Genetic analysis in Excel. Population genetic software for teaching and research. Mol. Ecol. Notes.

[30] Yeh F.C., Yang R.C., Boyle T.B., Ye Z.H., Mao J.X. (1997). POPGENE, the User-Friendly Shareware for Population Genetic Analysis.

[31] Agapow P.M., Burt A. (2001). Indices of multilocus linkage disequilibrium. Mol. Ecol. Notes.

[32] Excoffier L., Smouse P.E., Quattro J.M. (1992). Analysis of molecular variance inferred from metric distances among DNA haplotypes: Application to human mitochondrial DNA restriction data. Genetics.

[33] Halkett F., Plantegenest M., Prunier-Leterme N., Mieuzet L., Delmotte F., Simon J.C. (2005). Admixed sexual and facultatively asexual aphid lineages at mating sites. Mol. Ecol..

[34] Comont G., Corio-Costet M., Larignon P., Delmotte F. (2010). AFLP markers reveal two genetic groups in the French population of the grapevine fungal pathogen *Phaeomoniella chlamydospora*. Europ J. Plant Pathol..

[35] Durán A., Gryzenhout M., Drenth A., Slippers B., Ahumada R., Wingfield B.D., Wingfield M.I. (2010). AFLP analysis reveals a clonal population of *Phytophthora pinifolia* in Chile. Fungal Biol..

[36] Giraud T., Fortini D., Levis C., Leroux P., Brygoo Y. (1997). RFLP markers show genetic recombination in *Botryotinia fuckeliana* (*Botrytis cinerea*) and transposable elements reveal two sympatric species. Mol. Biol. Evol..

[37] Linde C.C., Zala M., Ceccarelli S., McDonald B.A. (2003). Further evidence for sexual reproduction in *Rhynchosporium secalis* based on distribution and frequency of mating-type alleles. Fungal Genet. Biol..

[38] Kerenyi Z., Moretti A., Waalwijk C., Olah B., Hornok L. (2004). Mating type sequences in asexually reproducing *Fusarium* species. Appl. Environ. Microbiol..

[39] Paoletti M., Rydholm C., Schwier E.U., Anderson M.J., Szakacs G., Lutzoni F., Debeaupuis J., Latge J., Denning D.W., Dyer P.S. (2005). Evidence for sexuality in the opportunistic fungal pathogen *Aspergillus fumigatus*. Curr. Biol..

[40] Milgroom M.G., Sotirovski K., Risteski M., Talbot Brewer M. (2009). Heterokaryons and parasexual recombinants of *Cryphonectria parasitica* in two clonal populations in southeastern Europe. Fungal Genet. Biol..

[41] Linde C., Drenth A., Kemp G.H.J., Wingfield M.J., von Broembsen S.L. (1997). Population structure of *Phytophthora cinnamomi* in South Africa. Phytopathology.

[42] Ivors K.L., Garbelotto M., Vries I.D.E., RuyterSpira C., Hekkert B., Rosenzweig N., Bonants P.J.M. (2006). Microsatellite markers identify three lineages of *Phytophthora ramorum* in US nurseries, yet single lineages in US forest and European nursery populations. Mol. Ecol..

[43] Prospero S., Hansen E.M., Grunwald N.J., Winton L.M. (2007). Population dynamics of the sudden oak death pathogen *Phytophthora ramorum* in Oregon from 2001 to 2004. Mol. Ecol..

