# Supplementary material for: Diversity of Genetic and Vegetative Compatibility Group of Colletotrichum coccodes Isolates from Chile Using Amplified Fragment Length Polymorphism Markers
Source: J Fungi (Basel). 2024 Mar 6;10(3):200. doi: 10.3390/jof10030200 (PMC10970801; doi:10.3390/jof10030200)
Supplement: Supplementary file 1 [file jof-10-00200-s001.zip › jof-2850547 -suppl/Supplementary Figure S1.pdf]

Supplementary Figure S1. Consensus tree for 148 C. coccodes isolates used in this study. The tree was executed using WinBoot software with 1000 bootstraps.
The numbers at the forks show the percentage of times the group consisting of the species which are to the right of that fork occurred

```
                                        +----C19-VCG1
                         +----------100.0
                         |              +----C60-VCG1
                         |
                    +-59.5              +----NE4-1-VCG1
                    |    |         +100.0
                    |    |    +-90.5    +----SCRI-C3
                    |    |    |    |
                    |    +-94.6    +---------T3-2
               +-90.5         |
               |    |         |         +----R-LAKE-22iL
               |    |         +-----100.0
               |    |                   +----SCRI-C2
          +-83.8    |
          |    |    +------------------------C138-VCG1
          |    |
          |    |                        +----11-2
     +-32.4    +--------------------100.0
     |    |                             +----13-9
     |    |
     |    |                             +----T7-8
     |    |                        +100.0
     |    +---------------------72.3    +----T7-2
     |                             |
+-10.8                             +---------T7-6
|    |
|    |                   +------------------T19-3
|    |                   |
|    |              +-39.9         +---------13-2
|    |              |    |    +-83.1
```

```
                              |    |              |    |    |    |    +----6-1
                              |    |              |    +-28.4    +100.0
                              |    +-----------56.8         |         +----T3-4
                              |                   |         |
                              |                   |         +--------------6-3
                         +--6.1                   |
                         |    |                   +------------------------9-9
                         |    |                                       +----8-4
                         |    |                             +------95.9
                         |    |                             |         +----1-1
                         |    |                        +100.0
                         |    |                        |    |         +----T10-8
                         |    |                        |    |    +100.0
                         |    |                        |    +-90.5    +----11-5
                         |    |                        |         |
                         |    +---------------------31.1         +---------T3-7
                         |                             |
                         |                             |              +----MN29-VCG2
                         |                             |         +100.0
                         |                             |    +-52.7    +----MT11-VCG2
                         |                             |    |    |
                         |                             +-79.1    +---------T10-3
                         |                                  |
+----------------------9.5                                  +--------------11-6
|                        |
|                        |                             +-------------------9-7
|                        |                             |
|                        |                             |              +----10-5
|                        |                   +------16.9         +-45.3
|                        |                   |         |         |    +----4-1
|                        |                   |         |    +-49.3
|                        |                   |         |    |    |    +----T1-7
|                        |                   |         +-89.2    +-86.5
|                        |                   |              |         +----5-6
```

```
     |                        |                   |              |
     |                        |                   |              +--------------T10-6
     |                        |              +--6.8
     |                        |              |    |                   +---------T7-1
     |                        |              |    |              +-95.3
     |                        |              |    |              |    |    +----T10-10
     |                        |              |    |         +-50.7    +-93.9
     |                        |              |    |         |    |         +----8-5
     |                        |              |    |    +-60.1    |
     |                        +------------2.7    |    |    |    +--------------T1-4
     |                                       |    +-29.7    |
     |                                       |         |    +------------------T10-4
     |                                       |         |
     |                                       |         +------------------------T10-7
     |                                       |
     |                                       |                        +---------COLL236-VCG3
     |                                       +---------------------57.4
     |                                                                |    +----COLL95iL-VCG3
     |                                                                +100.0
     |                                                                     +----COLL239iL-VCG3
     |
     |                                                                +---------ALB14-VCG2
     |                                                           +100.0
     |                                                           |    |    +----MT16-VCG2
     |                                                      +-93.2    +-97.3
     |                                                      |    |         +----12-1
     |                        +--------------------------78.4    |
     |                        |                             |    +--------------13-6
     |                        |                             |
     |                        |                             +------------------T1-7
     |                        |
     |                        |    +--------------------------------------------12-3
     |                        |    |
     |                        |    |                             +--------------T1-1
+-10.8                        |    |                             |
```

```
|    |                        |    |                   +------35.1         +----Si-23
|    |                        |    |                   |         |    +100.0
|    |                   +-71.6    |                   |         +-41.2    +----T7-5
|    |                   |    |    |                   |              |
|    |                   |    |    |              +-26.4              +---------T3-9
|    |                   |    |    |              |    |
|    |                   |    |    |              |    |                   +----Si-45
|    |                   |    |    |              |    |    +----------100.0
|    |                   |    |    |              |    |    |              +----8-2
|    |                   |    |    |              |    +-16.2
|    |                   |    |    |              |         |    +--------------5-4
|    |                   |    |    |              |         +-40.5
|    |                   |    |    |         +-40.5              |    +---------Aus136/07k
|    |                   |    |    |         |    |              +-99.3
|    |                   |    +100.0         |    |                   |    +----Si-51
|    |                   |         |         |    |                   +-64.9
|    |                   |         |         |    |                        +----ORG1-VCG4
|    |                   |         |         |    |
|    |                   |         |         |    |                        +----Aus-118
|    |                   |         |         |    |              +------86.5
|    |                   |         |         |    |              |         +----Aus59/07Ta
|    |                   |         |         |    +-----------83.1
|    |                   |         |         |                   |    +---------SA-H1
|    |              +-40.5         |         |                   +-73.6
|    |              |    |         |    +-25.0                        |    +----Si-22
|    |              |    |         |    |    |                        +-73.6
|    |              |    |         |    |    |                             +----Nad1-VCG5
|    |              |    |         |    |    |
|    |              |    |         |    |    |                             +----Aus88/07AH
|    |              |    |         |    |    |                   +------99.3
|    |              |    |         |    |    |                   |         +----Aus104/07A
|    |              |    |         |    |    |              +100.0
|    |              |    |         |    |    |              |    |    +---------SA-K1
|    |              |    |         +100.0    |              |    +-64.2
|    |         +-47.3    |              |    |         +-29.1         |    +----Si-25
```

```
     |    |         |    |    |              |    |         |    |         +-81.1
     |    |         |    |    |              |    |         |    |              +----WI-17-VCG5
     |    |         |    |    |              |    +------14.2    |
     |    |         |    |    |              |              |    +-------------------Nad3-VCG5
     |    |         |    |    |              |              |
     |    |         |    |    |              |              +------------------------C124-VCG4
     |    |    +-22.3    |    |              |
     |    |    |    |    |    |              +---------------------------------------MT5-VCG5
+-37.8    |    |    |    |    |
|    |    |    |    |    |    +------------------------------------------------------C114iL-VCG2
|    |    +--6.1    |    |
|    |         |    |    +-----------------------------------------------------------ORG2-VCG5
|    |         |    |
|    |         |    +----------------------------------------------------------------5-2
|    |         |
|    |         +---------------------------------------------------------------------NV02-24-1-VCG2
|    |
|    |                                                                          +----T6-2
|    |                                                                     +-95.9
|    |                                                           +------77.0    +----11-2
|    |                                                           |         |
|    |                                                           |         +---------T19-8
|    |                                                           |
|    |                                  +---------------------28.4              +----T6-1
|    |                                  |                        |    +------41.9
|    |                                  |                        |    |         +----5-7
|    |                                  |                        |    |
|    |                                  |                        +-70.3         +----T19-10
|    |                                  |                             |    +100.0
|    |                                  |                             |    |    +----T19-4
|    |                                  |                             +-53.4
|    |                                  |                                  |    +----T1-5
|    |                                  |                                  +100.0
|    |                                  |                                       +----T1-10
|    |                             +-12.2
|    |
```

```
     |    |                             |    |    +---------------------------------------T10-1
     |    |                             |    |    |
     |    |                             |    |    |                                  +----C46iL-VCG2
     |    |                             |    |    |              +----------------87.2
     |    |                             |    |    |              |                   +----10-1
     |    |                             |    |    |              |
     |    |                             |    |    |              |                   +----T7-4
     |    |                             |    |    |         +-72.3              +100.0
     |    |                             |    |    |         |    |         +-51.4    +----7-4
     |    |                             |    |    |         |    |         |    |
     |    |                             |    |    |         |    |    +-89.9    +---------T1-9
     |    |                             |    +-10.1         |    |    |    |
     |    |                             |         |         |    +-60.1    +--------------T19-3
     |    |                             |         |         |         |
+-19.6    |                             |         |         |         |    +--------------11-8
|    |    |                             |         |    +-49.3         +-99.3
|    |    |                             |         |    |    |              |    +---------9-8
|    |    +--------------------------12.8         |    |    |              +-58.1
|    |                                  |         |    |    |                   |    +----9-5
|    |                                  |         |    |    |                   +-89.9
|    |                                  |         |    |    |                        +----T3-5
|    |                                  |         |    |    |
|    |                                  |         |    |    |              +--------------T19-5
|    |                                  |         +-14.2    +-----------87.2
|    |                                  |              |                   |    +---------11-1
|    |                                  |              |                   +100.0
|    |                                  |              |                        |    +----11-4
|    |                                  |              |                        +-70.3
|    |                                  |              |                             +----11-3
|    |                                  |              |
|    |                                  |              |                             +----T6-7
|    |                                  |              +--------------------------54.7
|    |                                  |                                            +----7-5
|    |                                  |
|    |                                  |                                            +----8-3
```

```
     |    |                                  +-----------------------------------------56.8
     |    |                                                                                +----T10-9
     |    |
     |    |                                                                     +--------------C501-VCG7
     |    |                                                      +----------100.0
     |    |                                                      |              |    +---------C55iL-VCG7
     |    |                                                      |              +100.0
     |    |                                                      |                   |    +----NE-43-VCG6
     |    |                                                      |                   +100.0
     |    |                                                      |                        +----MN02-1-13-VCG6
     |    |                                                 +-73.6
     |    |                                                 |    |                   +---------SCRI-33
     |    |                                                 |    |    +----------100.0
     |    |                                                 |    |    |              |    +----C54iL-VCG7
     |    |                                                 |    |    |              +100.0
     |    |                                                 |    |    |                   +----NE-40-VCG6
     |    |                                                 |    |    |
     |    |                                                 |    +-83.8                   +----T6-8
     |    |                                                 |         |              +-98.6
     |    |                                                 |         |              |    +----T6-4
     |    |                                                 |         |    +------56.1
+-12.2    +---------------------------------------------100.0         |    |         |    +----T7-7
|    |                                                      |         |    |         +-81.1
|    |                                                      |         +-89.9              +----T7-8
|    |                                                      |              |
|    |                                                      |              |    +-------------SA-R13
|    |                                                      |              +-96.6
|    |                                                      |                   |    +---------NE-02-45-VCG6
|    |                                                      |                   +-69.6
|    |                                                      |                        |    +----NE02-41-VCG6
|    |                                                      |                        +100.0
|    |                                                      |                             +----452-VCG6
|    |                                                      |
|    |                                                      +----------------------------------T19-6
|    |
```

```
     |    |                                                                               +---------T7-9
     |    |                                                      +---------------------16.9
     |    |                                                      |                        |    +----10-2
     |    |                                                      |                        +-27.7
     |    |                                                      |                             +----6-7
     |    |                                                      |
     |    |                                                 +--6.1                             +----7-7
     |    |                                                 |    |    +---------------------43.9
     |    |                                                 |    |    |                        +----13-1
     |    |                                                 |    |    |
     |    |                                                 |    |    |         +-------------------T6-6
     |    |                                                 |    +-22.3         |
     |    |                                                 |         |    +-56.1    +--------------14-1
     |    |                                                 |         |    |    |    |
     |    |                                                 |         |    |    +-79.1         +----5-3
     |    |                                                 |         |    |         |    +-48.0
+-32.4    |                                                 |         +-59.5         +-68.9    +----11-7
|    |    |                                            +--2.7              |              |
|    |    |                                            |    |              |              +---------13-7
|    |    |                                            |    |              |
|    |    |                                            |    |              |                   +----T3-6
|    |    |                                            |    |              +---------------100.0
|    |    |                                            |    |                                  +----T3-3
|    |    |                                            |    |
|    |    |                                            |    |                                  +----9-6
|    |    |                                            |    |                   +-----------29.1
|    |    |                                            |    |                   |              +----9-10
|    |    |                                            |    |                   |
|    |    +------------------------------------------7.4    +----------------10.1         +---------8-6
|    |                                                 |                        |    +-48.0
|    |                                                 |                        |    |    |    +----12-2
|    |                                                 |                        +-47.3    +100.0
|    |                                                 |                             |         +----12-8
|    |                                                 |                             |
|    |                                                 |                             +--------------T6-5
```

```
 |    |                                                 |
 |    |                                                 |                             +--------------T3-8
 |    |                                                 |                        +-26.4
 |    |                                                 |                        |    |    +---------T10-2
 |    |                                                 |                        |    +-57.4
 |    |                                                 +---------------------19.6         |    +----13-8
 |    |                                                                          |         +-93.2
 |    |                                                                          |              +----T3-1
 |    |                                                                          |
 |    |                                                                          +-------------------9-4
 |    |
 |    +----------------------------------------------------------------------------------------------T3-10
 |
 |                                                                                              +----T1-8
 |                                                                                         +100.0
 |                                                                                         |    +----T1-6
 +--------------------------------------------------------------------------------------62.8
                                                                                           |    +----C501iL-VCG7
                                                                                           +100.0
                                                                                                +----MN02-1-14-VCG6
```

WinBoot computational run time: 0:01:32.984
