# Supplementary material for: Diversity of Genetic and Vegetative Compatibility Group of Colletotrichum coccodes Isolates from Chile Using Amplified Fragment Length Polymorphism Markers
Source: J Fungi (Basel). 2024 Mar 6;10(3):200. doi: 10.3390/jof10030200 (PMC10970801; doi:10.3390/jof10030200)
Supplement: Supplementary file 1 [file jof-10-00200-s001.zip › jof-2850547 -suppl/Supplementary Table S1.docx]

Supplementary Table S1. Detailed information for *Colletotrichum coccodes* isolates amplified using the internal transcribed spacer and their accession numbers on National Center for Biological Information (NCBI).

|  | **Country** | **Location** | | **Variety** | **Isolate code** | **Accession number** |
| --- | --- | --- | --- | --- | --- | --- |
| 1 | Chile | Coñab, Achao, Chiloe Island | | Michuña roja | 1.1 | JX294031.1 |
| 2 | Chile | Nueva Brauna, Puerto Montt | | Rosara | 4.1 | JX294017.1 |
| 3 | Chile | Coñab, Achao, Chiloe Island | | Michuña negra | 5.2 | JX293998.1 |
| 4 | Chile | Coñab, Achao, Chiloe Island | | Michuña negra | 5.3 | JX293969.1 |
| 5 | Chile | Coñab, Achao, Chiloe Island | | Michuña negra | 5.4 | JX293951.1 |
| 6 | Chile | Coñab, Achao, Chiloe Island | | Michuña negra | 5.6 | JX293973.1 |
| 7 | Chile | Coñab, Achao, Chiloe Island | | Michuña negra | 5.7 | JX293992.1 |
| 8 | Chile | Coñab, Achao, Chiloe Island | | Clavela | 6.1 | JX294042.1 |
| 9 | Chile | Coñab, Achao, Chiloe Island | | Clavela | 6.2 | JX294029.1 |
| 10 | Chile | Coñab, Achao, Chiloe Island | | Clavela | 6.7 | JX293970.1 |
| 11 | Chile | Coñab, Achao, Chiloe Island | | Murta | 7.4 | JX294022.1 |
| 12 | Chile | Coñab, Achao, Chiloe Island | | Murta | 7.5 | JX293977.1 |
| 13 | Chile | Coñab, Achao, Chiloe Island | | Murta | 7.6 | JX294026.1 |
| 14 | Chile | Coñab, Achao, Chiloe Island | | Pie | 8.2 | JX293950.1 |
| 15 | Chile | Coñab, Achao, Chiloe Island | | Pie | 8.3 | JX293971.1 |
| 16 | Chile | Coñab, Achao, Chiloe Island | | Pie | 8.4 | JX294043.1 |
| 17 | Chile | Coñab, Achao, Chiloe Island | | Pie | 8.5 | JX293991.1 |
| 18 | Chile | Coñab, Achao, Chiloe Island | | Pie | 8.6 | JX294012.1 |
| 19 | Chile | Coñab, Achao, Chiloe Island | | Desirée | 9.4 | JX294006.1 |
| 20 | Chile | Coñab, Achao, Chiloe Island | | Desirée | 9.5 | JX294041.1 |
| 21 | Chile | Coñab, Achao, Chiloe Island | | Desirée | 9.6 | JX294002.1 |
| 22 | Chile | Coñab, Achao, Chiloe Island | | Desirée | 9.7 | JX293985.1 |
| 23 | Chile | Coñab, Achao, Chiloe Island | | Desirée | 9.8 | JX294035.1 |
| 24 | Chile | Coñab, Achao, Chiloe Island | | Desirée | 9.9 | JX294037.1 |
| 25 | Chile | Coñab, Achao, Chiloe Island | | Desirée | 9.10 | JX294016.1 |
| 26 | Chile | Coñab, Achao, Chiloe Island | | Desirée | 10.1 | JX294039.1 |
| 27 | Chile | Coñab, Achao, Chiloe Island | | Desirée | 10.2 | JX294024.1 |
| 28 | Chile | Coñab, Achao, Chiloe Island | | Desirée | 10.3 | - |
| 29 | Chile | Coñab, Achao, Chiloe Island | | Romano | 11.1 | JX293987.1 |
| 30 | Chile | Coñab, Achao, Chiloe Island | | Romano | 11.2 | JX294044.1 |
| 31 | Chile | Coñab, Achao, Chiloe Island | | Romano | 11.3 | JX293978.1 |
| 32 | Chile | Coñab, Achao, Chiloe Island | | Romano | 11.4 | JX293981.1 |
| 33 | Chile | Coñab, Achao, Chiloe Island | | Romano | 11.5 | JX294011.1 |
| 34 | Chile | Coñab, Achao, Chiloe Island | | Romano | 11.6 | JX294038.1 |
| 35 | Chile | Coñab, Achao, Chiloe Island | | Romano | 11.7 | JX293966.1 |
| 36 | Chile | Coñab, Achao, Chiloe Island | | Romano | 11.8 | JX294036.1 |
| 37 | Chile | Remehue, Osorno | | Yagana | 12.1 | JX293957.1 |
| 38 | Chile | Remehue, Osorno | | Yagana | 12.2 | JX294008.1 |
| 39 | Chile | Remehue, Osorno | | Yagana | 12.3 | JX293999.1 |
| 40 | Chile | Remehue, Osorno | | Yagana | 12.8 | JX294003.1 |
| 41 | Chile | Remehue, Osorno | | Desirée | 13.1 | JX294014.1 |
| 42 | Chile | Remehue, Osorno | | Desirée | 13.2 | JX294030.1 |
| 43 | Chile | Remehue, Osorno | | Desirée | 13.4 | - |
| 44 | Chile | Remehue, Osorno | | Desirée | 13.5 | JX293994.1 |
| 45 | Chile | Remehue, Osorno | | Desirée | 13.6 | JX294004.1 |
| 46 | Chile | Remehue, Osorno | | Desirée | 13.7 | JX294009.1 |
| 47 | Chile | Remehue, Osorno | | Desirée | 13.9 | JX294033.1 |
| 48 | Chile | Rapacoa, La Union | | Desirée | 14.1 | JX294010.1 |
| 49 | Chile | Remehue, Osorno | | Yagana | T1.1 | JX293954.1 |
| 50 | Chile | Remehue, Osorno | | Yagana | T1.4 | JX293967.1 |
| 51 | Chile | Remehue, Osorno | | Yagana | T1.5 | JX294027.1 |
| 52 | Chile | Remehue, Osorno | | Yagana | T1.6 | JX293958.1 |
| 53 | Chile | Remehue, Osorno | | Yagana | T1.7 | JX293988.1 |
| 54 | Chile | Remehue, Osorno | | Yagana | T1.8 | JX293962.1 |
| 55 | Chile | Remehue, Osorno | | Yagana | T1.9 | JX293979.1 |
| 56 | Chile | Remehue, Osorno | | Yagana | T1.10 | JX293976.1 |
| 57 | Chile | Remehue, Osorno | | Asterix | T3.1 | JX293964.1 |
| 58 | Chile | Remehue, Osorno | | Asterix | T3.2 | JX293956.1 |
| 59 | Chile | Remehue , Osorno | | Asterix | T3.3 | - |
| 60 | Chile | Remehue, Osorno | | Asterix | T3.4 | JX294028.1 |
| 61 | Chile | Remehue, Osorno | | Asterix | T3.5 | JX294034.1 |
| 62 | Chile | Remehue, Osorno | | Asterix | T3.6 | JX293975.1 |
| 63 | Chile | Remehue, Osorno | | Asterix | T3.7 | JX294001.1 |
| 64 | Chile | Remehue, Osorno | | Asterix | T3.8 | JX293989.1 |
| 65 | Chile | Remehue, Osorno | | Asterix | T3.9 | JX293952.1 |
| 66 | Chile | Remehue, Osorno | | Asterix | T3.10 | JX293968.1 |
| 67 | Chile | Remehue, Osorno | | Karú | T6.1 | JX293995.1 |
| 68 | Chile | Remehue, Osorno | | Karú | T6.2 | JX294013.1 |
| 69 | Chile | Remehue, Osorno | | Karú | T6.4 | JX293959.1 |
| 70 | Chile | Remehue, Osorno | | Karú | T6.5 | JX294018.1 |
| 71 | Chile | Remehue, Osorno | | Karú | T6.6 | JX293974.1 |
| 72 | Chile | Remehue, Osorno | | Karú | T6.7 | JX293982.1 |
| 73 | Chile | Remehue, Osorno | | Karú | T6.8 | JX293986.1 |
| 74 | Chile | Remehue, Osorno | | Pukará | T7.1 | JX294015.1 |
| 75 | Chile | Remehue, Osorno | | Pukará | T7.2 | JX293997.1 |
| 76 | Chile | Remehue, Osorno | | Pukará | T7.4 | JX294023.1 |
| 77 | Chile | Remehue, Osorno | | Pukará | T7.5 | JX293953.1 |
| 78 | Chile | Remehue, Osorno | | Pukará | T7.6 | JX294032.1 |
| 79 | Chile | Remehue, Osorno | | Pukará | T7.7 | JX293960.1 |
| 80 | Chile | Remehue, Osorno | | Pukará | T7.8 | JX294000.1 |
| 81 | Chile | Remehue, Osorno | | Pukará | T7.9 | JX294021.1 |
| 82 | Chile | Remehue, Osorno | | Patagonia | T10.1 | JX294020.1 |
| 83 | Chile | Remehue, Osorno | | Patagonia | T10.2 | JX293955.1 |
| 84 | Chile | Remehue, Osorno | | Patagonia | T10.3 | JX293993.1 |
| 85 | Chile | Remehue, Osorno | | Patagonia | T10.4 | JX293972.1 |
| 86 | Chile | Remehue, Osorno | | Patagonia | T10.5 | JX294019.1 |
| 87 | Chile | Remehue, Osorno | | Patagonia | T10.6 | JX294007.1 |
| 88 | Chile | Remehue, Osorno | | Patagonia | T10.7 | JX293965.1 |
| 89 | Chile | Remehue, Osorno | | Patagonia | T10.8 | JX294025.1 |
| 90 | Chile | Remehue, Osorno | | Patagonia | T10.9 | JX293963.1 |
| 91 | Chile | Remehue, Osorno | | Cardinal | T10.10 | JX294005.1 |
| 92 | Chile | Remehue, Osorno | | Cardinal | T19.3 | JX293980.1 |
| 93 | Chile | Remehue, Osorno | | Cardinal | T19.4 | JX294040.1 |
| 94 | Chile | Remehue, Osorno | | Cardinal | T19.5 | JX293984.1 |
| 95 | Chile | Remehue, Osorno | | Cardinal | T19.6 | JX293990.1 |
| 96 | Chile | Remehue, Osorno | | Cardinal | T19.7 | JX293961.1 |
| 97 | Chile | Remehue, Osorno | | Cardinal | T19.8 | JX293983.1 |
| 98 | Chile | Remehue, Osorno | | Cardinal | T19.9 | - |
| 99 | Chile | Remehue, Osorno | | Cardinal | T19.10 | JX293996.1 |
| 100 | USA | - | | - | C19-VCG1 | [JX293923.1](https://www.ncbi.nlm.nih.gov/nuccore/JX293923.1) |
| 101 | USA | - | | - | C138-VCG1 | [JX293924.1](https://www.ncbi.nlm.nih.gov/nuccore/JX293924.1) |
| 102 | USA | - | | - | NE4-1-VCG1 | [JX293925.1](https://www.ncbi.nlm.nih.gov/nuccore/JX293925.1) |
| 103 | USA | - | | - | R-LAKE-22iL-VCG1 | [JX293926.1](https://www.ncbi.nlm.nih.gov/nuccore/JX293926.1) |
| 104 | USA | - |  | - | ALB14-VCG2 | [JX293927.1](https://www.ncbi.nlm.nih.gov/nuccore/JX293927.1) |
| 105 | USA | - | | - | MT16-VCG2 | [JX293928.1](https://www.ncbi.nlm.nih.gov/nuccore/JX293928.1) |
| 106 | USA | - | | - | C114iL-VCG2 | [JX293929.1](https://www.ncbi.nlm.nih.gov/nuccore/JX293929.1) |
| 107 | USA | - | | - | NV02-24-1-VCG2 | [JX293930.1](https://www.ncbi.nlm.nih.gov/nuccore/JX293930.1) |
| 108 | USA | - | | - | COLL236-VCG3 | [JX293931.1](https://www.ncbi.nlm.nih.gov/nuccore/JX293931.1) |
| 109 | USA | - | | - | COLL95iL-VCG3 | [JX293932.1](https://www.ncbi.nlm.nih.gov/nuccore/JX293932.1) |
| 110 | USA | - | | - | COLL239iL-VCG3 | [JX293933.1](https://www.ncbi.nlm.nih.gov/nuccore/JX293933.1) |
| 111 | USA | - | | - | C124-VCG4 | [JX293934.1](https://www.ncbi.nlm.nih.gov/nuccore/JX293934.1) |
| 112 | USA | - | | - | ORG1-VCG4 | [JX293935.1](https://www.ncbi.nlm.nih.gov/nuccore/JX293935.1) |
| 113 | USA | - | | - | Nad1-VCG5 | [JX293936.1](https://www.ncbi.nlm.nih.gov/nuccore/JX293936.1) |
| 114 | USA | - | | - | WI-17-VCG5 | [JX293937.1](https://www.ncbi.nlm.nih.gov/nuccore/JX293937.1) |
| 115 | USA | - | | - | MT5-VCG5 | [JX293938.1](https://www.ncbi.nlm.nih.gov/nuccore/JX293938.1) |
| 116 | USA | - | | - | ORG2-VCG5 | [JX293939.1](https://www.ncbi.nlm.nih.gov/nuccore/JX293939.1) |
| 117 | USA | - | | - | Nad3-VCG5 | [JX293940.1](https://www.ncbi.nlm.nih.gov/nuccore/JX293940.1) |
| 118 | USA | - | | - | 452-VCG6 | [JX293941.1](https://www.ncbi.nlm.nih.gov/nuccore/JX293941.1) |
| 119 | USA | - | | - | NE02-41-VCG6 | [JX293942.1](https://www.ncbi.nlm.nih.gov/nuccore/JX293942.1) |
| 120 | USA | - | | - | NE-02-45-VCG6 | [JX293943.1](https://www.ncbi.nlm.nih.gov/nuccore/JX293943.1) |
| 121 | USA | - | | - | MN02-1-13-VCG6 | [JX293944.1](https://www.ncbi.nlm.nih.gov/nuccore/JX293944.1) |
| 122 | USA | - | | - | MN02-1-14-VCG6 | [JX293945.1](https://www.ncbi.nlm.nih.gov/nuccore/JX293945.1) |
| 123 | USA | - | | - | NE-40-VCG6 | [JX294049.1](https://www.ncbi.nlm.nih.gov/nuccore/JX294049.1) |
| 124 | USA | - | | - | NE-43-VCG6 | [JX294050.1](https://www.ncbi.nlm.nih.gov/nuccore/JX294050.1) |
| 125 | USA | - | | - | C55iL-VCG7 | [JX293946.1](https://www.ncbi.nlm.nih.gov/nuccore/JX293946.1) |
| 126 | USA | - | | - | C501iL-VCG7 | [JX293947.1](https://www.ncbi.nlm.nih.gov/nuccore/JX293947.1) |
| 127 | USA | - | | - | C54iL-VCG7 | [JX293948.1](https://www.ncbi.nlm.nih.gov/nuccore/JX293948.1) |
| 128 | USA | - | | - | C501-VCG7 | [JX293949.1](https://www.ncbi.nlm.nih.gov/nuccore/JX293949.1) |
| 129 | USA | - | | - | C60-VCG1 | [JX294045.1](https://www.ncbi.nlm.nih.gov/nuccore/JX294045.1) |
| 130 | USA | - | | - | MT11-VCG2 | [JX294046.1](https://www.ncbi.nlm.nih.gov/nuccore/JX294046.1) |
| 131 | USA | - | | - | MN29-VCG2 | [JX294047.1](https://www.ncbi.nlm.nih.gov/nuccore/JX294047.1) |
| 132 | USA | - | | - | C46iL-VCG2 | [JX294048.1](https://www.ncbi.nlm.nih.gov/nuccore/JX294048.1) |
| 133 | South Africa | - | | - | SA-H1 | - |
| 134 | South Africa | - | | - | SA-K14 | [JX294051.1](https://www.ncbi.nlm.nih.gov/nuccore/JX294051.1) |
| 135 | South Africa | - | | - | SA-R13 | - |
| 136 | Scotland | - | | - | SCRI-C3 | [JX294052.1](https://www.ncbi.nlm.nih.gov/nuccore/JX294052.1) |
| 137 | Scotland | - | | - | SCRI-C2 | [JX294053.1](https://www.ncbi.nlm.nih.gov/nuccore/JX294053.1) |
| 138 | Scotland | - | | - | SCRI-33 | [JX294054.1](https://www.ncbi.nlm.nih.gov/nuccore/JX294054.1) |
| 139 | Netherland | - | | - | Si-45 | [JX294055.1](https://www.ncbi.nlm.nih.gov/nuccore/JX294055.1) |
| 140 | Netherland | - | | - | Si-23 | [JX294056.1](https://www.ncbi.nlm.nih.gov/nuccore/JX294056.1) |
| 141 | Netherland | - | | - | Si-51 | [JX294057.1](https://www.ncbi.nlm.nih.gov/nuccore/JX294057.1) |
| 142 | Netherland | - | | - | Si-22 | [JX294058.1](https://www.ncbi.nlm.nih.gov/nuccore/JX294058.1) |
| 143 | Netherland | - | | - | Si-25 | [JX294059.1](https://www.ncbi.nlm.nih.gov/nuccore/JX294059.1) |
| 144 | Australia | - | | - | Aus136/07k | [JX294060.1](https://www.ncbi.nlm.nih.gov/nuccore/JX294060.1) |
| 145 | Australia | - | | - | Aus59/07Ta | [JX294061.1](https://www.ncbi.nlm.nih.gov/nuccore/JX294061.1) |
| 146 | Australia | - | | - | Aus104/07AH | [JX294062.1](https://www.ncbi.nlm.nih.gov/nuccore/JX294062.1) |
| 147 | Australia | - | | - | Aus-118 | [JX294063.1](https://www.ncbi.nlm.nih.gov/nuccore/JX294063.1) |
| 148 | Australia | - | | - | Aus88/07AH | [JX294064.1](https://www.ncbi.nlm.nih.gov/nuccore/JX294064.1) |
